# “Homozygous, and compound heterozygous mutation in 3 Turkish family with Jervell and Lange-Nielsen syndrome: case reports”

**DOI:** 10.1186/s12881-017-0474-8

**Published:** 2017-10-16

**Authors:** Fahrettin Uysal, Burcu Turkgenc, Guven Toksoy, Ozlem M. Bostan, Elif Evke, Oya Uyguner, Cengiz Yakicier, Hulya Kayserili, Ergun Cil, Sehime G. Temel

**Affiliations:** 1 Department of Pediatric Cardiology, University of Uludag, School of Medicine, Bursa, Turkey; 2 Acibadem Genetic Diagnostic Center, Istanbul, Turkey; 3 Department of Medical Genetics, Istanbul University, Faculty of Medicine, Istanbul, Turkey; 4 Department of Pediatric, Cardiology, Uludag University, Faculty of Medicine, Bursa, Turkey; 5 Bursa Genetic Diagnostic Center, Bursa, Turkey; 6 Department of Molecular Biology and Genetic, Acibadem University, Faculty of Science, Istanbul, Turkey; 7 Department of Medical Genetics, Koc University, Faculty of Medicine, Istanbul, Turkey; 8 Department of Histology& Embryology, Near East University, Faculty of Medicine, Nicosia, North Cyprus; 9 Department of Medical Genetics, Uludag University, Faculty of Medicine, Bursa, Turkey; 10 Department of Histology & Embryology, Uludag University, Faculty of Medicine, Bursa, Turkey; 11 Gorukle campuss, Uludag University, School of Medicine, 16059, Nilufer, Bursa, Turkey

**Keywords:** Jervell-Lange-Nielsen syndrome, Deafness, Homozygous or compound heterozygous mutations, Case report

## Abstract

**Background:**

Jervell and Lange-Nielsen syndrome (JLNS) isa recessive model of long QT syndrome which might also be related to possible hearing loss. Although the syndrome has been demonstrated to be originated from homozygous or compound heterozygous mutations in either the *KCNQ1* or *KCNE1* genes, additional mutations in other genetic loci should be considered, particularly in malignant course patients.

**Case presentations:**

Three patients were admitted into hospital due to recurrent seizures/syncope, intrauterine and postnatal bradycardia respectively; moreover all three patients had congenital sensorineural hearing-loss. Their electrocardiograms showed markedly prolonged QT interval. Implantable defibrillator was implanted and left cardiac sympathetic denervation was performed due to the progressive disease in case 1. She had countless ventricular fibrillation and appropriate shock while using an implantable defibrillator. The DNA sequencing analysis of the *KCNQ1* gene disclosed a homozygous c.728G > A (p.Arg243His) missense mutation in case1. Further targeted next generation sequencing of cardiac panel comprising 68 gene revealed a heterozygous c.1346 T > G (p.Ile449Arg) variant in *RYR2* gene and a heterozygous c.809G > A (p.Cys270Tyr) variant in *NKX2–5* gene in the same patient. Additional gene alterations in *RYR2* and *NKX2–5* genes were thought to be responsible for progressive and malignant course of the disease.

As a result of DNA sequencing analysis of *KCNQ1* and *KCNE1* genes, a compound heterozygosity for two mutations had been detected in *KCNQ1* gene in case 2: a maternally derived c.477 + 1G > A splice site mutation and a paternally derived c.520C > T (p.Arg174Cys) missense mutation. Sanger sequencing of *KCNQ1* and *KCNE1* genes displayed a homozygous c.1097G > A (p.Arg366Gln) mutation in *KCNQ1* gene in case 3. β-blocker therapy was initiated to all the index subjects.

**Conclusions:**

Three families of JLNS who presented with long QT and deafness and who carry homozygous, or compound heterozygous mutation in *KCNQ1* gene were presented in this report. It was emphasized that broad targeted cardiac panels may be useful to predict the outcome especially in patients with unexplained phenotype-genotype correlation. Clinical presentations and molecular findings will be discussed further to clarify the phenotype genotype associations.

## Background

Long QT syndrome (LQTS) is a cardiac disease characterized by an abnormal repolarization in cardiac cells causing a prolonged QT interval on the electrocardiogram (ECG). Ventricular tachycardia known as “torsades de pointes” is one of the main features of LQTS and leads to seizures, syncopes, cardiac arrest and sudden death [1].

LQTS has two inherited forms. The extensive form is the autosomal dominant condition referred to as Romano-Ward syndrome (RWS), which most commonly presents itself in the form of cardiac associated symptoms [2, 3]. The less common form is Jervell and Lange-Nielsen Syndrome (JLNS), inherited as a recessive manner and presents a severe cardiac phenotype in addition to profound bilateral hearing-loss [4]. Most of the causative genes identified for LQTSs encode the ion channels that are involved in the regulation of the cardiac action potential period, these genes include the Na^+^and K^+^ channel genes; *SCN5A, HERG*, *KCNE2*, *KCNQ1* and *KCNE1*. JLNS usually results from recessive bi-allelic mutations leading loss of function in either the *KCNQ1* or *KCNE1* genes [5, 6].

These voltage-gated channels characteristically contain four subunits that surround a pore which is centrally localized. Each subunit is composed of six transmembrane segments (S1–S6) including the NH2 and COOH termini which is located on the intracellular membrane. The voltage sensor of the channel is S4 segment. A P-loop domain connects the S5-S6 segment to create the pore zone. Also, the transmembrane domains of S2–S3 and S4–S5 are connected by Cytoplasmic loops (C-loops) [7]. It was reported that missense mutations and mutations localized at the transmembrane part were related to high risk for cardiolocigal events than the mutations localizing at the C-terminal region. Furthermore, it was suggested that C-loops influence the regulation of adrenergic channel by Protein Kinase A (PKA) [8].

Over the past decade some isolated cases of JLNS presenting with only cardiac manifestations without hearing loss (so called AR LQT1) has been reported. It has been suggested that hearing preservation in these KCNQ1 homozygotes or compound heterozygotes is derived from the presence of mildly effective mutations that can not totally remove the Kv7.1 (KvLQT1) function and maintain the normal K^+^ cycle with the occurence of sufficient residual IKs current in the inner ear, however the normal electrical activity of the heart is disrupted [9].

Long QT syndromes may often be misdiagnosed as epilepsy due to the associated seizures that occur [10, 11]. In the current report, we identify both clinical and genetic findings from three proband’s with JLNS, and discuss the phenotype-genotype correlation with each case.

## Case presentations

### Case 1 (family-I)

**Fig. 1:**
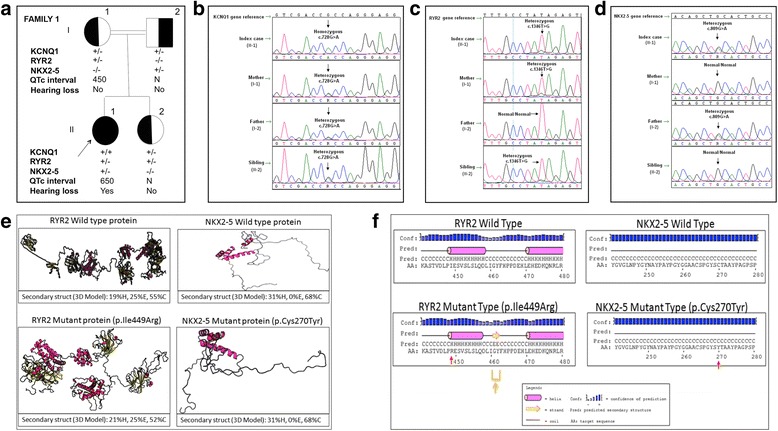
The molecular and clinical findings for family I (**a**) Pedigree of the family I; Case 1 (II-1) who is carrying homozygous c.728G > A (p.Arg243His) mutation in the *KCNQ1* gene, heterozygous c.1346 T > G (p.Ile449Arg) alteration in *RYR2* gene and heterozygous c.809G > A (p.Cys270Tyr) alteration in *NKX2–5* gene and the carrier status of the other family members were shown in the fig. (N: Normal, +: Mutant allele, −: Normal allele) (**b**) DNA sequencing image for KCNQ1 mutation (**c**) DNA sequencing image for RYR2 variant (**d**) DNA sequencing image for NKX2–5 variant (**e**) Predicted secondary structures and 3D modelling of wild type and mutant RYR2 protein (left) and NKX2–5 protein (right) (H: Helix, E: Beta Sheet, C: Loop). For 3-state secondary structure, It is predicted that the secondary structure of RYR2 mutant type is altered when compared to wild type and the secondary structure of NKX2–5 mutant type is not altered when compared to wild type (**f**) Predicted secondary structures of wild type and mutant RYR2 protein (left) and NKX2–5 protein (right). The altered residues of RYR2 (p.Ile449Arg) and NKX2–5 (p.Cys270Tyr) are shown with red arrows. It is predicted that the secondary structure of RYR2 mutant type is altered when compared to wild type (shown with orange arrow) and the secondary structure of NKX2–5 mutant type is not altered when compared to wild type

The initial findings of this case (Fig. 1a; II-1) have previously been published in Pediatric Cardiology [12].

The first molecular analysis of the female patient revealed a homozygous c.728G > A (p.Arg243His) mutation in the *KCNQ1* gene (NM_000218.2, NP_000209.2) as a consequence of consanguineous marriage (Fig. 1b). The patient was diagnosed with JLNS. She was also diagnosed with refractory epilepsy and treated with anti-epileptic drugs in her previous hospitalizations.

Later on, during one of the patient’s follow-up visits, according to the Implantable cardioverter-defibrillator (ICD) record, it was revealed that two electric shocks were delivered and the patient was monitored in the intensive care unit. On the 4th day in the intensive care unit, the patient experienced ventricular fibrillation 90 times which resulted in ICD battery replacement and left cervical sympathectomy. Five additional proper electric shocks were delivered after sympathectomy. The phenotype of the proposita changed dramatically, because of the additive malignant symptoms, the patient was screened for 68 hereditary arrhythmia gene panel (Ion Ampliseq Custom Panel comprising 68 gene associated with arrhythmias) to search for the additional clinically related gene variants.

Further investigations revealed a heterozygous c.1346 T > G (p.Ile449Arg, rs373331669) alteration in the *RYR2* gene (NM_001035.2, NP_001026.2) with unknown pathogenicity in the SNP database (http://www.ncbi.nlm.nih.gov/projects/SNP) and lower minor allele frequency (MAF) of 0.0000083 in Exac Browser database (http://exac.broadinstitute.org/gene/ENSG00000198626). Furthermore a heterozygous c.809G > A (p.Cys270Tyr, rs587782931) alteration in the *NKX2–5* gene (NM_004387.3*,*NP_004378.1) was listed as a likely benign allele in the SNP database and ClinVar database (http://www.ncbi.nlm.nih.gov/clinvar/variation/156161/) with lower MAF of 0,000073 (http://exac.broadinstitute.org/gene/ENSG00000183072). Parental investigations demonstrated that the *RYR2* variant was maternally and *NKX2–5* variant was paternally inherited (Fig. 1c and d). For these variants, we used the four prediction algorithms (SIFT (http://sift.jcvi.org/www/SIFT_enst_submit.html), PolyPhen-2 (http://genetics.bwh.harvard.edu/pph2/), PROVEAN (http://provean.jcvi.org/seq_submit.php) and Align-GVGD (http://agvgd.hci.utah.edu/agvgd_input.php) to estimate the potential function sof the mutant protein. The *RYR2* gene variant (c.1346 T > G) was presumed as a deleterious allele on the strength of the in-silico prediction tools. The *NKX2–5* gene variant was predicted as benign with respect to PolyPhen-2 and was regarded as a damaging allele according to SIFT, PROVEAN and Align-GVGD. Conservation of residues was investigated across several species running UCSC GenomeBrowser (https://genome.ucsc.edu/cgi-bin/hgBlat) and PolyPhen-2. The analysis results showed that the variation regions of *RYR2* and *NKX2–5* are not highly conserved among species. RaptorX (http://raptorx.uchicago.edu/StructurePrediction) and PSIPRED (http://bioinf.cs.ucl.ac.uk/psipred/) were used for in-silico analyses to examine the effects of the *RYR2* and *NKX2–5* variations on the secondary structures of proteins. When the secondary and 3-dimensional (3D) structures of the proteins were compared, it was evident that the beta-sheet, helix, and loop structures were disrupted in the RYR2 mutated protein when compared to the wild-type protein. But, there was no apparent distinction in terms of the secondary structure between the NKX2–5 mutant protein and the wild-type protein (Fig. 1e and f).

### Case 2 (family II)

**Fig. 2:**
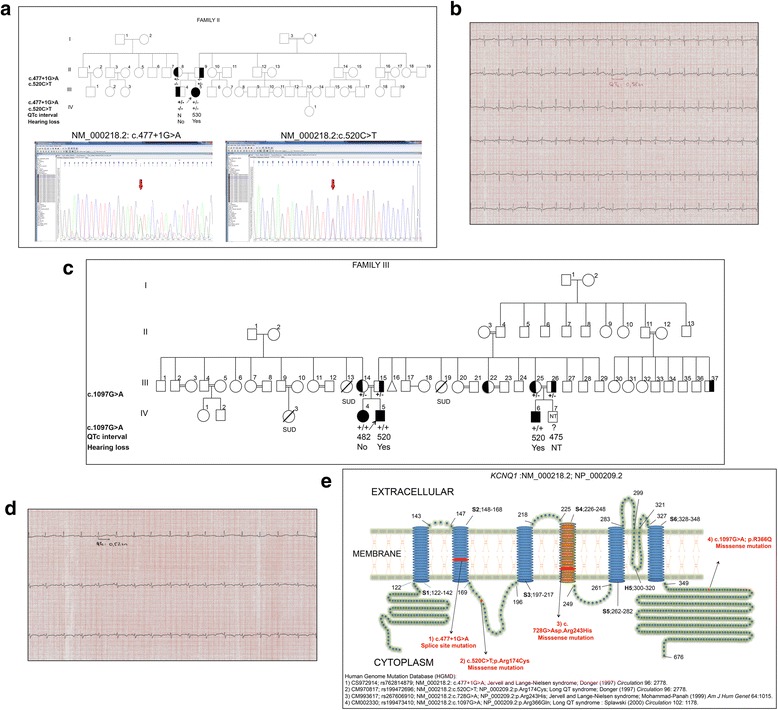
The molecular and clinical findings for family II and family III, and KCNQ1 mutations illustrated on KCNQ1 protein (**a**) Pedigree of the family II; Case 2 (III-5) who is carrying compound heterozygous mutations of c.477 + 1G > A and c.520C > T (p.Arg174Cys) in *KCNQ1* gene and the carrier status of the other family members were shown above, (N = Normal, +: Mutant allele, −: Normal allele), DNA sequencing images of the mutations in the proband were presented below (**b**) The ECG of the proband (III-5) in family II showing the prolongation of QTc (530 ms) interval (**c**) Pedigree of the family III; Case 3 (IV-5) who is carrying the c.1097G > A (p.R366Q) mutation in *KCNQ1* gene and the carrier status of the other family members were presented (N = Normal, SUD = Sudden unidentified death, NT = Not tested, +:Mutant allele, −:Normal allele) (**d**) The ECG of the proband (IV-5) in family III showing the prolongation of QTc (520 ms) (**e**) The illustration showing all of the *KCNQ1* mutations seen in our three cases (S1, S2, S3, S5,S6: Helical, transmembranic domains; S4:Helical, Voltage-sensor, transmembranic domain; H5: Pore-forming, intramembranic domain)

A 33-day-old female infant (Fig. 2a, III-5) was examined due to intrauterine bradycardia. She was the first child of a nonconsanguineous marriage. Bilateral deafness was confirmed by a brainstem evoked response audiometry (BERA) test and her routine biochemical laboratory test results were within normal range.

No family history of convulsion and sudden cardiac death for three generations was evident.

She had undergone cochlear implant surgery. Her resting 12 lead ECG showed prominent prolongation in QTc distance of 530 ms (Fig. 2b). Propranolol was commenced at a dose of 0.004 g/kg four times in a day. The ECG of both parents displayed normal resting ECG readings.

Her follow-up visits for 3.5 years did not show any visible signs of syncope or adverse event.

The mutation analysis was conducted using the Sanger sequencing method of the coding exons and exon intron junctions of *KCNQ1* (NM_000218.2, NP_000209.2) and *KCNE1* (NM_000219.5, NP_000210.2) genes.

The molecular genetic analysis of the patient disclosed compound heterozygosity for splice site (c.477 + 1G > A) and missense (c.520C > T, p.Arg174Cys) mutations in the *KCNQ1* gene (Fig. 2a). Heterozygous c.477 + 1G > A mutation was identified in the maternal allele, and heterozygous c.520C > T (p.Arg174Cys) mutation was identified in the paternal allele.The elder brother (III:4) was also heterozygous for the c.520C > T (p.Arg174Cys) mutation and had a normal QT interval.

### Case 3 (family III)

A 5 year old male child (Fig. 2c; IV-5) was examined due to pre-diagnosis of long QT syndrome. He was a second child of a first-degree consanguineous marriage. He displayed symptoms of tachypnea and postnatal bradycardia and was transferred to another hospital on the second day of his birth.

In the physical examination, bilateral hearing loss was determined. His resting 12 lead ECG exhibited a prolongation in QTc distance of 520 ms (Fig. 2d). The ECG of both parents demonstrated normal ECG readings at rest. No syncope was detected on his follow-up period. Propranolol was commenced at a dose of 0.0015 g/kg four times in a day.

The mutation analysis of proposita was carried out by Sanger sequencing of the coding exons and exon intron connections of *KCNQ1* (NM_000218.2, NP_000209.2) and *KCNE1* (NM_000219.5, NP_000210.2) genes. The molecular genetic analysis disclosed homozygosity for c.1097G > A (p.Arg366Gln) mutation in the *KCNQ1* gene (Fig. 2c). The parents were identified to have this mutation in heterozygous state.

His elder sister (Fig. 2c, IV:4) was evaluated for physical examination. She was not reported to have any previous syncope attacks and her hearing was normal. Her resting 12 lead ECG showed prolongation in QTc distance of 482 ms. Atenolol was commenced at a dose of 0.0006 g/kg two times in a day. She was carrying the c.1097G > A (p.Arg366Gln) mutation in homozygous form. Proposita’s 3 month old brother (Fig. 2c, IV:7), was also invited for clinical observation. His resting 12 lead ECG showed prolongation in QTc interval of 475 ms and bilateral deafness was also detected in his physical examination. Propranolol was administered at a dose of 0.0005 g/kg four times in a day. Clinical investigation commenced upon the identification of the same *KCNQ1* genotype as proposita.

His cousin (Fig. 1c, IV:6), a 3 year-old male was also hospitalized because of a convulsive attack. He was the first son of a first-degree consanguineous marriage by normal delivery. His first electroencephalogram was dysrhythmic which later on was normal. Bilateral deafness was determined through a physical examination. His resting 12 lead ECG exhibited prolongation in QTc distance of 520 ms. The ECG of his mother (III:25) and father (III:26) showed normal ECG. Atenolol was acommenced at a dose of 0.0006 g/kg two times in a day. He was also diagnosed with refractory epilepsy and treated with anti-epileptic drugs in his previous hospitalizations. Molecular genetic analysis revealed the same mutation in homozygous form as proposita and parental investigation revealed the heterozygous status.

## Discussion & conclusion

Long QT syndrome may often be a fatal disease, and symptomatic cases who do not receive treatment have a elevated mortality ratio, 21% within a year from the initial attack [13]. JLNS is featured by congenital bilateral sensorineural deafness and long QTc distance that is generally higher than 500 ms [14].

*KCNQ1* and *KCNE1* gene mutations have been demonstrated to be associated with JLNS [15]. *KCNQ1* encodes a subunit of voltage-gated K^+^ channel protein which have ainteraction with the β subunit encoded by the *KCNE1* gene to generate a cardiac K^+^ channel. The molecular genetic analysis JLNS families demonstrated that the homozygous/compound heterozygous mutations in the *KCNQ1* gene are the main cause of the syndrome.

The c.728G > A (p. Arg243His) mutation of case 1 (II-1 of family I)-was reported previously in two non-consanguineous French families in a compound heterozygous manner who displayed a typical JLNS and in two consanguineous Turkish JLNS families [12, 15–17]. This missense mutation caused the change of the 243th basic charged polar arginine to histidine that has similar physicochemical properties. It is positioned in S4 domain of the protein (Fig. 2e). Mutations striking to S4/S5 domains are predicted to disrupt the gating function of the ion channels and alter *KCNQ1* interactions with the minK sub-units [18]. Chouabe et al. demonstrated that this mutation is related to a considerably shorter prolongation of the QTc distance, though it may cause sudden deaths due to full loss of function [17]. It seems clearly that the cardiac functions is affected by the *KCNQ1* mutations, based on either the voltage shift and loss of function in the ion channel. The voltage alteration mutations may also end up with inconstant phenotypes. The homozygous carriers of p.Arg243His mutation have the the maximum shift of the voltage current in the cardiac cells, however the heterozygous carriers are asymptomatic with no major longer OTc interval. The cardiac symptoms of case 1 (II-1 of family I) became progressively severe in an age dependent fashion and ICD implantation and cervical sympathectomy was performed due to recurrent ventricular tachycardia attacks. Due to the progressive and malignant disease course, further targeted next generation sequencing of cardiac panel containing 68 functional genes were examined and the patient had additional heterozygous alterations in the *RYR2* and *NKX2–5* genes. The *RYR2* gene had been previously described to be involved in catecholaminergic polymorphic ventricular tachycardia type 1 (CPVT1) and arrhythmogenic right ventricular dysplasia type 2 (ARVD2) (OMIM#600996 and 604772) but the patient phenotype was consistent with neither CPVT1 nor ARVD2. Although these two genes were not associated with LQTS or JLNS alone, they could aggravate the clinical phenotype of the patient additive to the homozygous *KCNQ1* mutation.

The c.477 + 1G > A splice site and the c.520C > T (p. Arg174Cys) missense mutations detected in family II were both described previously in North America and Europe, [19, 20]. As far as we know, this is the first report of compound heterozygote Turkish family of JLNS carrying these mutations. The splice site mutation detected in case2 (III-5 of family II) is located in intron 2. Recently, Zehelein J. and his colleagues reported that this mutation alters the evolutionally conserved splice donor site resulting to splice error. Predicted exon skipping introduces a premature translational termination resulting in truncated KCNQ1 potassium channel subunits [21].

In a functional study examining a p. Arg174Cys mutation demonstrated that PKA, protein kinase C (PKC) and Phosphatidylinositol-4, 5-bisphosphate (PIP2) regulation decreased [22]. This study also revealed that a decline in the activation of either PKA or PKC may promote to the disease phenotype by reducing the activation of normal ion channel via receptor stimulus.

The c.1097G > A (p. Arg366Gln) mutation detected in family III was first reported in RWS and recently in one Turkish JLNS family [23–25]. The mutation was found at the C-terminal of *KCNQ1.* It was reported that the mutant p. Arg366Gln channel presents a weaker interference with PIP2 in contrast with the wild type channel. An increasement in the channel activation was observed for the two C-terminal *KCNQ1* mutants of p. Arg366Gln and p. Arg555Cys. These C terminal mutants might be protective and reduce the severity of the mutation [23]. Cases with pathogenic variants at the C terminal part of the *KCNQ1* subunit was found to be less prone to cardiological events than cases with pathogenic variants in the transmembrane parts [26]. In family III, all affected subjects had a milder QTc prolongation and milder cardiac phenotype. Interestingly one of the subjects (IV: 4 of family III) had no hearing loss, although she had the same homozygous mutation as the index case (IV-5 of family III) and the other affected cousin (IV-6 of family III). It remains largely elusive that what determines this great variability in the severity of disorder, where even a relative carrying the same pathogenic variant demonstrate significant differences in phenotypic/clinical manifestations. Giudicessi JR et al. suggested that a great majority of unrelated KCNQ1 homozygotes and compound heterozygotes with a possible autosomal recessive model of inheritance (64%, 7 out of 11) show no sensorineural deafness. Their study showed that the full breakdown of KvLQT1 mediated K^+^ secretion in the cochlea linked to the biallelic inheritance of 2 truncating/haploid insufficient pathogenic variants (complete loss-of-function) is the major mechanism that affects the homeostasis of inner ear fluid, causing the downfall of endolymph in the inner ear and sensorineural hearing loss in JLNS cases and murine models of JLNS [9]. Missense variations with dominant-negative effects on the tetrameric KCNQ1 channel results in an altered molecular function but not complete loss of function. Recent electrophysiological studies showed that homozygous and compound heterozygous pathogenic variants in either *KCNQ1* or *KCNE1* gene involved in JNLS resulted in a broad range of spectrum of effects from loss of function to dominant negative behavior [27]. Recently Bhuiyan ZA et al., reported a homozygous novel splice site c.387-5 T > A mutation in the first intron of the *KCNQ1* gene, in the two Saudi Arabian families. RNA analysis demonsrated that c.387-5 T > A mutation leads incomplete transcriptional error of the *KCNQ1* gene, leaving 10% of the normal transcript intact, which restores the auditory function. An other intronic pathogenic variant leading to incomplete exon 2 skipping in *KCNQ1* saves hearing function in JLNS [28]. Apparently, as in our family III, the homozygous c.1097G > A (p. Arg366Gln) mutation does not cause a complete loss of KvLQT1 function in endolymph of the inner ear in all of the homozygous carriers. The amount of residual KvLQT1 function necessary for auditory preservation in JLNS is not known. The other mechanism proposed to explain the development of intact hearing in this case could be the mosaicism. It can be speculated that JLNS mutation mosaicism results in functional channels in the marginal cells of the inner ear with ordinary membrane transmission, so the phenotypic outcomes are not monitored in the inner ear. Although the possibility seemed unlikely in familial cases, it still cannot be ruled out. Another speculation is that there may be other potential genetic factors in normal functioning of cochlear hair cells leading to intact hearing.

Therefore, whole genome exome sequencing may assist with the investigation of the underlying genetic causes of normal hearing. Furthermore, to explain this variability in the clinical phenotype; expression studies of wild-type and mutant type *KCNQ1* using cardiac muscle cells [29] and cochlear hair cells obtained from patient specific induced pluripotent stem cells need to be performed.

Children with JLNS are frequently misdiagnosed with epilepsy at the beginning of therapy, causing both delayed care for JLNS and improper anti-epileptic medication [10]. In our families; Case 1 (II-1 of family I) and the cousin of case 3 (IV-6 of family III) were also diagnosed as refractory epilepsy and treated with anti-epileptic drugs in their previous hospitalizations. In the current study the two cases underlined the importance of careful examination of the patient having seizures and primarily to exclude the cardiac cause for the differential diagnosis. This would not only save the lives of the patients but will also assist with proper treatments and specific genetic counseling sessions for other high-risk individuals in their families.

However, a group of these cases are presumably to have a neurocardiac syndrome through ion channels, as recent evidence demonstrated that epileptiform activity determined with electroencephalography (EEG) in 15% of LQTS cases who showed seizures or seizure like crisis [30]. Therefore, because of this possible coexistence of both conditions we routinely began to do detailed cardiologic and neurologic screening of patients at the University of Uludag.

Management of JLNS consists of β-blockers, ICD and/or left cardiac sympathetic denervation (LCSD) for those with β-blocker resistive symptoms for the cardiological events, and cochlear implants for the recovery of hearing loss [4]. Beta-blocker therapy is the fundamental medication for JLNS; potential ICD and/or LCSD for those with β-blocker resistive symptoms, insufficiency to take β-blockers, and/or cardiac arrest history [31]. Beta-blockers significantly decrease the sudden death risk.

Beta blocker theraphy is clinically indicated in all symptom-free cases meeting diagnostic criteria, consisting of those who possess a mutation on genetic screening and a normal QTc interval [4]. Usually, implantation of ICD is not indicated for asymptomatic cases of JLNS. Prophylactic ICD treatment might be conceived for cases with JLNS who show no symptoms but suspected to be at very high-risk (e.g., those with at least 2 pathogenic mutations on genetic testing and/or family history of young sudden unexplained death) [32].

Therefore, we advise that all carriers of an identified pathogenic variant in either the *KCNQ1* or the *KCNE1* gene be thoroughly examined at a minimum by favour of ECG and clinical family history, before initiating prophylactic therapy like beta-blockers in these cases. Cardiac screening is recommended in more complex cases. Because the mutation carriers of JLNS might have a slight deafness sign, a hearing test should also be considered. Medications and conditions that stimulate the prolongation on the QT interval such as dynamic sport activities, horror films, loud environments are to be avoided. Training and educating of all family members for cardiac arrest resuscitation might be life saving.

After all, here we present three families of JLNS who display long QT and deafness.

This genotype-phenotype study suggests that the patients with trans-membrane versus C-terminus mutations and these with mutations having dominant negative versus haploinsufficiency demonstrate the variable degree of the failure of ion channels as well as the clinical course of the disorder.

It is well known that protein function is affected by the C terminal mutations in *KCNQ1* gene far less degree. Also, it is precise that there is no absolute lack of K^+^ inflow into the endolymph of the inner ear in our JLNS patient IV: 4 of family III with intact hearing who is carrying a homozygous C-terminal c.1097G > A (p. Arg366Gln) missense mutation. Our study suggests a milder effect of the mutation on the clinical phenotype for family III as well as hearing preservation of the IV: 4 patient of family III. The degree of the remaining Kv7.1 function necessary for auditory preservation in KCNQ1 homozygosity and/or compound heterozygosity is not known.

It was also emphasized that broad targeted cardiac panels or whole exome analysis may be useful to predict the outcome especially in patients with unexplained phenotype-genotype correlation according to the mutation detected and the progressive and malignant course.

Our research group screened approximately 200 cases with our custom made 68 gene cardiac panel diagnosed as hereditary arrhythmias like recessive JLNS and/or LQT syndromes, we have seen that this disease group is highly heterogeneous in terms of genetics and caused by mutations of more than one variant and/or multiple variants [33]. Due to the genetic complexity of hereditary arrhythmias like LQTS, JLNS, it currently makes it necessary to screen the other cardiac genes in addition to the most common genes related to the complex phenotypes before the molecular-genetic workup is completed. Families, clinicians and researchers together will have a essential role in the advancement of biomedicine towards personalized treatments.

## Abbreviations

ARVD2: Arrhythmogenic right ventricular dysplasia type 2
BERA: Brainstem evoked response audiometry
C-loops: Cytoplasmic loops
CPVT1: Catecholaminergic polymorphic ventricular tachycardia type 1
ECG: Electrocardiogram
EEG: Electroencephalography
ICD: Implantable cardioverter-defibrillator
JLNS: Jervell and Lange-Nielsen syndrome
Kv7.1 (KvLQT1): Voltage-Gated Potassium Channel Subunit
LCSD: left cardiac sympathetic denervation
LQTS: Long QT syndrome
MAF: Minor allele frequency
Ms.: milliseconds
OMIM: Online Mendelian Inheritance in Man
PIP2: Phosphatidylinositol-4, 5-bisphosphate
PKA: Protein kinase A
PKC: Protein kinase C
RWS: Romano-Ward syndrome
